# Advances in the Electrophysiological Recordings of Long-Term Potentiation

**DOI:** 10.3390/ijms24087134

**Published:** 2023-04-12

**Authors:** Feixu Jiang, Stephen Temitayo Bello, Qianqian Gao, Yuanying Lai, Xiao Li, Ling He

**Affiliations:** 1 Department of Neuroscience, City University of Hong Kong, Kowloon, Hong Kong; 2 Research Institute of City University of Hong Kong, Shenzhen 518057, China

**Keywords:** LTP, iLTP, electrophysiological experiments, field potential recording, single-cell potential recording, astrocytes, gliotransmitters, tripartite synapses

## Abstract

Understanding neuronal firing patterns and long-term potentiation (LTP) induction in studying learning, memory, and neurological diseases is critical. However, recently, despite the rapid advancement in neuroscience, we are still constrained by the experimental design, detection tools for exploring the mechanisms and pathways involved in LTP induction, and detection ability of neuronal action potentiation signals. This review will reiterate LTP-related electrophysiological recordings in the mammalian brain for nearly 50 years and explain how excitatory and inhibitory neural LTP results have been detected and described by field- and single-cell potentials, respectively. Furthermore, we focus on describing the classic model of LTP of inhibition and discuss the inhibitory neuron activity when excitatory neurons are activated to induce LTP. Finally, we propose recording excitatory and inhibitory neurons under the same experimental conditions by combining various electrophysiological technologies and novel design suggestions for future research. We discussed different types of synaptic plasticity, and the potential of astrocytes to induce LTP also deserves to be explored in the future.

## 1. Introduction

Neuronal cells and glial cells are the main components of the brain. Approximately 50% of the brain is neuronal cells; the other half is glial cells, which all play an important role in the mammalian brain [1,2,3,4]. Billions of neurons are connected and communicate via synapses inextricably linked to behavior, memory, and neurological diseases. Synaptic plasticity is a change in neural connection strength that occurs in response to activity [5]. Reorganization of the structural and functional connections of synapses occurs in response to internal or external stimuli, leading to the strengthening or weakening of synaptic connections via synaptic plasticity [6,7]. Long-term potentiation (LTP) has been widely used as an ideal model for studying synaptic plasticity, learning, and memory [7,8,9]. 

**Figure 1:**
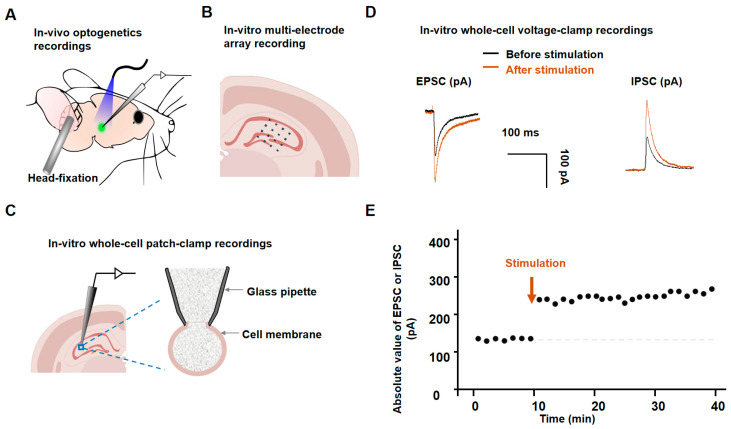
Three types of electrophysiological recordings in long-term potentiation (LTP). (**A**): In vivo optogenetics recordings. (**B**): In vitro electrode array recording. (**C**): In vitro whole-cell patch-clamp recordings. (**D**,**E**): Traces and normalized slope of excitatory postsynaptic potential or inhibitory postsynaptic current EPSC/IPSC before and after stimulation.

LTP is a classical synaptic plasticity caused by the persistent stimulation-induced enhancement of neuronal signaling. Specifically, when neurons receive this series of stimulations, subsequent single-pulse stimulations cause an enhanced and prolonged excitatory postsynaptic potential (EPSP) or inhibitory postsynaptic current (IPSC) in the postsynaptic population potential [10]. A brief stimulation can induce LTP for periods ranging from a few minutes to several months, and this persistent state of LTP differentiates it from other forms of synaptic plasticity [11]. Although different brain regions exhibit different forms of LTP and different mediators can modulate LTP, the signature of LTP activity data does not change (as shown in Figure 1D,E). LTP is triggered by postsynaptic depolarization of the cell membrane and elevated calcium concentration.

In vivo extracellular recordings [12], microelectrode arrays [13], and patch clamps [14] are the most common electrophysiological techniques used to detect LTP [6,7,8]. As a result, the data utilized to assess LTP formation may also be divided into the field and individual potentials. Field potentials were identified using in vivo extracellular recordings and microelectrode arrays, whereas individual potentials were detected using the patch clamp. The first recording of LTP activity in neuronal cells was conducted using microelectrode arrays in the hippocampus (HP) of mice [15]. However, in recording the acute plasticity induction protocol of excitatory or inhibitory synapses, they only recorded a single type of neuron action potentiation with LTP (Table 1). As mentioned above, the recordings of detection or comparison of the firing states of these two types of neurons (excitatory and inhibitory) were limited at the same time and space. 

Here, we raise the following three questions:

1. Why did most researchers use field potential to detect LTP at excitatory synapses while using the single-cell potential to detect LTP at inhibitory synapses, respectively?
2. What is the mechanism of LTP at the inhibitory synapses? Is this similar to excitatory synapses?
3. Do LTP and LTP of inhibition (iLTP) occur independently? What does an inhibitory neuron do while excitatory neurons are stimulated to induce LTP?

**Table 1:** The timeline and recordings of long-term potentiation studies.

Year	Mechanism or Event	Induction	Recording Method	Brain Area	Ref.
1970–1980	Discovery of LTP	10–20 Hz 100 Hz	Extracellular micro-electrodes Population EPSP	HP, CA1, CA3	[10]
	Brain slice recording on LTP	3–50 Hz	Population EPSP	HP, CA1	[16]
	LTP needs synaptic transmission	100 Hz	Population EPSP	HP	[17]
	Ca^2+^- dependent	100 Hz	Extracellular population spike EPSP	HP, CA1	[18]
1980–1990	NMDAR Postsynaptic Ca^2+^	>35 Hz	Extracellular recording	HP, CA1	[19,20,21,22]
	Activation of NMDA receptors blocks GABAergic inhibition	Tetanic electrical stimuli	Extra and intracellular recording IPSP	HP, CA1	[23]
	LTP needs NMDAR	HFS	Intracellular recording	Visual cortex	[24]
1990–2000	Single-cell recording EPSP-spike	HFS	Intracellular recording	CA1	[25]
	GABA_B_R regulates NMDA to induce LTP	0.5–100 Hz	Monosynaptic inhibitory pathway IPSC	HP, granule cells	[26,27]
	Induction of LTP needs mGluRs	HFS 100 Hz	Extracellular field potentials Whole-cell patch clamp	HP, CAl CA3	[28,29]
	NMDAR dependent Ca^2+^	100 Hz	Field potentials EPSP	HP	[30]
	NO mediate LTP	100 Hz	Field potentials EPSP	HP	[31]
	GABA AR Independent	HFS 50 Hz	Intracellular recording IPSP	visual cortex (LV)	[32]
	GABA_B_R dependent Ca^2+^ Release	HFS 50 Hz	Intracellular and whole-cell recording IPSP/IPSC	visual cortex (LV)	[33]
	NMDA-dependent inhibition	100 Hz	Whole-cell and extracellular recording	HP, CA1	[34]
	Bi-directional plasticity	100 Hz	Intracellular recording	HP	[35]
	NO mediate LTP	50 Hz	Whole-cell ruptured patch recording EPSCs	HP	[36]
	GABAergic synaptic LTP	0.1 Hz	Intracellular recording	Neonatal rats, HP	[37]
2000–2010	mGluR GABA B R Postsynaptic Ca^2+^	TBS	Whole-cell recording IPSP	HP CA1	[38]
	NMDAR-nondependent	HFS (30 Hz)	Whole-cell recording IPSP and EPSP	Lateral Amygdala	[39]
	The pairing of presynaptic activity with sub-threshold postsynaptic depolarization Postsynaptic	50 Hz postsynaptic depolarization-60 mV	Patch clamp IPSC	Visual Cortex(LIV)	[40]
	GABA AR NO initiates iLTP NMDA-independent	HFS	Whole-cell patch clamp IPSC	VTA	[41]
	BDNF-TrkB	HFS (50 Hz)	Whole-cell patch clamp Voltage clamp IPSC	Visual cortex (LV)	[42,43]
	mGluR5 postsynaptic calcium, NMDAR- nondependent	TBS	Whole-cell patch clamp EPSP	The visual cortex, L II/III	[44]
	BDNF and cAMP-dependent PKA	LFS (0.05 Hz)	Patch clamp–Voltage clamp IPSP and IPSC	HP, CA3	[45]
	Astrocyte-induced independent-LTP	0.5 Hz	Whole-cell patch clamp	HP, CA1, CA3	[46]
	D-serine from astrocytes activates NMDAR	100 Hz	Whole-cell recordings	HP	[47,48]
2010–2020	BDNF-TrkB	50 Hz	Whole-cell patch clamp IPSC	Auditory Cortex (AC)	[49]
	Cholecystokinin (CCK) modulates the plasticity of GABA Synapses	HFS	Whole-cell recording IPSC	Dorsomedial Hypothalamus	[50]
	NMDAR triggers CCK release	HFS (100 Hz)	In vivo, fEPSP	AC	[51]
	Dual-channel optogenetic LTP-induction	Optogenetic HFS (oHFS) 50 Hz	Field potential recording fEPSP Whole-cell recording NMDAR/AMPA-EPSP	Dorsal striatum	[52]
	Astrocytic ATP is necessary for LTP_CCK_	HFS (100 Hz)	Whole-cell recording	Hypothalamus	[50]
	Small increase in Astrocytic ATP release	HFS (100 Hz)	Extracellular field recordings, fEPSP	HP, CA1	[53]
2020–2023	CCK Potentiates GABAergic Synapses	20 Hz	Whole-cell patch clamp	VTA	[54]
	Spatial regulation of excitatory and inhibitory synaptic plasticity	LFS 2 Hz, 4 Hz	Whole-cell patch clamp	HP	[55]
	Astrocyte dystrophy parallels impaired LTP	HFS 100 Hz	Patch clamp	HP, CA1	[56]
	Optogenetic induction of orbitostriatal LTP	oHFS 50 Hz	Whole-cell recording oEPSCs	Dorsomedial Striatum	[57]
	Novel CCKR: GPR173 Mediates iLTP	HFLS	In vivo extracellular and in vitro patch clamp	Neocortex	[58]
	Capacitive energy storage in the phospholipid bilayer	LFS 0.01 Hz	Patch clamp	DPhPC multilamellar vesicles (MLVs)	[59]

Several studies have focused on different aspects of LTP, the interplay of molecular mechanisms, the effect of different stimulation protocols (such as intensity and frequency), and the spike timing of LTP involved in its formation. Here, we focus on the most fundamental but rarely discussed analysis of the experimental electrophysiological data of LTP detection. In this review, we summarize the experimental electrophysiological data of LTP and iLTP in mammalian brains over the past 50 years in an attempt to answer the above three questions.

## 2. Field Potential and Single-Cell Potential Recording in LTP

We summarized the different recording methods used to detect LTP at excitatory and inhibitory synapses to answer the first question. Based on this research, we further attempted to analyze why only single-cell potential recording was applied to detect LTP at inhibitory synapses but not field potential recording.

### 2.1. Field Potential Recording at Excitatory Synapses

Since 1966, when LTP was first discovered by Lømo and was reported in the HP of rabbits by Lømo and Bliss et al. in 1973, an electrophysiological system capable of monitoring population EPSPs was used [10,15,60]. This system enhances synaptic strengths in specific brain regions. The electrophysiological system resulted in a 43% increase in the amplitude of the EPSP population, which represents the depolarization of granule cells. Population spike amplitude reduction signaling is the most common sign of potentiation. The 40% amplitude of the population spike represents cell firing. LTP occurs if one or two of the conditions mentioned above are satisfied. The earliest LTP model above lays the foundation for the subsequent study and modeling of synaptic plasticity. The potential mechanism of excitatory synaptic LTP was studied by detecting the field potential.

The studies of LTP in the past 50 years are shown in Table 1, which shows that most LTP studies have recorded population excitatory neuronal potentials, especially in the HP. These studies focused on exploring various LTP mechanisms, including NMDAR-dependent LTP [61], NMDAR-nondependent LTP [62], voltage-dependent Ca^2+^ channels [63], and NMDAR triggering CCK release to induce LTP [51], as evidenced by field potential result data. Lømo discovered LTP using this population cell recording, a simple and convenient system to capture excitatory neuronal firing activity. This experimental device for measuring field potentials in vivo (in vivo recording, Figure 1A) and brain slices (MED64 multi-electrode array technology, Figure 1B) illustrates that the population EPSP reflects the overall excitability of the neuronal population [1,51,64]. Some studies also recorded LTP at specific excitatory synapses using whole-cell patch clamps but not field potential recording [65,66,67]. Compared to field potential recording, the patch clamp can precisely record specific neuronal firing patterns [67,68]. In contrast, the field potential recording technique was used for recording excitatory neuronal LTP in certain brain areas. One of the reasons is researchers used a patch clamp to record inhibitory neuronal activity, which will be discussed in the following chapter.

The penetrating microelectrode mainly used in the research is the microwire type. This type of microelectrode is implanted in the brain to record neural activity action potentials (APs) and local field potentials (LFPs). The method can provide more information than non-penetrating microelectrodes. It has been used short-term and documented in rodent studies with low levels of tissue damage [69]. The most obvious advantage of in vivo extracellular recording is that it cannot only simultaneously record the electrical activity of many neurons in multiple brain regions but also allow the use of population enhancement data to detect the excitatory synaptic action of the population when the animal is in a more natural state. In vivo extracellular recording allows the study of the brain by stimulating and exploring the temporal and spatial connections between neuronal firing in different brain regions by analyzing the firing patterns of neurons, thus understanding the brain’s coding mechanisms in various tasks. Additionally, using MED64 Multi-electrode Array Technology for brain slice studies is more convenient than in vivo and patch-clamp techniques. The alignment allows for the precise geometric assignment of stimulus and recording locations.

However, owing to the non-uniform distribution of voltage-dependent channels in dendrites [2], the EPSP and IPSP signals cancel each other out during recording, which indicates that the EPSP results in the field potential recordings are the sum of the EPSP and IPSP after computational processing by the recording system, which are not unitary data. Therefore, the field potentials can only reflect the sum of local neuronal activity but cannot show single-cell firing when the population activity is triggered, much less the excitatory or inhibitory state of neurons simultaneously. Therefore, it is difficult for researchers to detect the firing states of excitatory and inhibitory neurons under the same experimental conditions.

Although excitatory synaptic LTP has been one of the most studied forms of neuroplasticity thus far and field potentials are convenient to reflect its activity, the limitations of single-field potential recordings have led to many questions that cannot be adequately answered, hence the many controversial theories of LTP. Recently, combining two-photon microscopy and fluorescent labeling techniques with electrophysiological experiments has provided evidence of presynaptic enhancement during LTP [63]. The combination of intracellular or whole-cell recordings elucidated NMDAR-dependent LTP dependent on increased postsynaptic Ca^2+^ concentrations [33,70,71,72], as well as presynaptic voltage-dependent Ca^2+^ channels [73], with pharmacological findings of multiple forms of LTP, such as mGluR-dependence [72].

Furthermore, early experimental techniques’ limitations disregarded the inhibitory synapses’ long-term plasticity. When researchers use the field potential detection technique to detect and analyze LTP in excitatory neurons, the question arises: what about inhibitory synapses? The answer to this question can be found in the single-cell potentiation recordings of γ-aminobutyric acid (GABA)ergic inhibitory synapses.

### 2.2. Extracellular Ionic Currents That Are of Dual Nature

Changes in extracellular ion concentration are produced within the central nervous system as part of normal neuronal activity, which can affect neuronal activity by altering cellular resting potentials [74]. Many studies focused on the brain’s extracellular potassium concentration ([K+]_0_) partially regulated by K^+^ spatial buffering by glial cells. Newman et al. detected the efflux of K^+^ from dissociated salamander Müller cells using ion-selective microelectrodes, a kind of field potential recording, in 1984 [74], and, in isolated frog retina, after treatment with aspartate, the photoinduced change in the extracellular potassium ions concentration [K+]_0_ was similar to slow PIII potential (sPIII), both increased in the whole range of light stimulus durations [75]. (Relationship between photoinduced changes in the intercellular concentration of potassium ions and transretinal potential generation by the Muller cells of the retina) Newman and Odette established a model simulating retinal processes based on the K^+^ hypothesis, producing the b-wave response [76]. According to this model, a realistic sPIII potential responding to [K+]_0_ decreases in the distal retina, and the K^+^ reproduces accurately [76]. Additionally, increasing in [K+]_0_ in vitreous humor was detected by double-barreled K^+^-selective microelectrodes, recorded from frog and mudpuppy eyecups after light-evoked potassium increasing within the retina [77].

Astrocytes are gradually regarded as excellent targeted therapeutic candidates for treating neurological diseases. Measuring astrocyte activity in the brain is rather important for neurologic development. Astrocyte activity has been detected in the low-frequency band < 1 Hz, while the standard models of recordings of extracellular potentials can only capture higher frequency potential [78]. Normally, researchers detect extracellular potentials by combining multicompartmental models showing neural electrodynamics and volume conductor theory, limited to simulating the slow components of extracellular potentials, which depend on ion concentration and the effect on extracellular diffusion potentials glial buffering currents [79]. To solve the problem, Marte et al. established an electrodiffusive neuron-extracellular-glia (edNEG) model, considered the first model combining compartmental neuron modeling with an electrodiffusive framework for intra- and extracellular ion concentration dynamics in a local piece of neuro-glial brain tissue [79]. In another study, the extracellular electrical activity of human astrocytes was successfully recorded by separating the signals received from human astrocytes cultured on a microelectrode array (MEA) into seven frequency bands [78]. 

### 2.3. Single-Cell Potential Recording at Inhibitory Synapses

#### 2.3.1. Intracellular Recordings

Artola and Singer used intracellular recordings to demonstrate that the activation threshold of the NMDA machinery, and, consequently, the susceptibility to LTP, was strongly influenced by inhibitory processes [24]. Activity-dependent plasticity of GABAergic synaptic transmission was studied in neonatal rat HP slices using intracellular recording techniques and illustrated that, during early development, bidirectional synaptic plasticity is expressed by GABA_A_ receptors and that activation (or inactivation) of NMDA receptors determines LTP-GABA_A_ induction [35]. 

#### 2.3.2. Patch Clamp: Whole-Cell Recording 

In the 1970s, patch-clamp techniques were introduced to the field of neuroscience to illustrate synaptic transmission and were then applied to LTP studies in 1987 [41,80,81,82]. Subsequently, in 1991, a combination of receptor antagonists and single-cell potential recording techniques demonstrated that GABA_B_ receptors could modulate NMDA release to regulate LTP [26,27], which gradually highlighted GABAR and GABAergic neurons.

As shown in Table 1, the study of iLTP in inhibitory neurons has been much slower than the rapid development of early excitatory neuronal LTP studies. iLTP is attributed to the increased diversity of relatively sparse GABAergic interneurons, in addition to the limitations of the previously used simple and convenient field potential recording method mentioned above [83,84,85], which fails to exhibit the same consistent reflective state to plasticity induction as tightly packed pyramidal neurons in randomly sampled extracellular field potential recordings [86]. The above recordings would lead to difficulty in inducing and detecting LTP in GABAergic cells. The advent of single-cell recordings has allowed for a more comprehensive study of inhibitory neurons. It has greatly improved our understanding of inhibitory cells owing to advances in experimental equipment and the diversity of methods.

Presently, the study of inhibitory neurons mainly relies on patch clamps because of the characteristics of GABAergic interneurons and the diversity of recording modes in the patch clamp. Patch-clamp systems include current and voltage clamps, allowing researchers to quickly change the stimulus and recording modes. Moreover, the unique patch-clamp whole-cell recording technique solves the problem of an extremely negative signal-to-noise ratio compared with traditional intracellular recording, which makes compensation very easy. Thus, the patch-clamp technique enables the separation of synaptic structures from the effects of mixed networks and allows studying brain slices under controlled environmental conditions. For example, it can stimulate specific pathways independently and record specific postsynaptic cells without polluting synaptic input from other connected brain regions [87]. In addition, information obtained from whole-cell recordings (especially brain slices or in vivo recordings) reflects changes in cellular function (and even intercellular messaging) coupled with the ease of changing the extracellular fluid environment. Therefore, whole-cell recordings are more suitable for pharmacological studies of ion channels.

## 3. LTP Mechanisms of Excitatory and Inhibitory Synapses

Consistent increases in neurotransmitter release result in omnipresent forms of LTP [88]. Plenty of evidence suggests that neuronal activity can trigger sustained increases in neurotransmitter release at excitatory and inhibitory synapses, leading to LTP. The use of intracellular and patch-clamp recordings revealed various interesting mechanisms that trigger inhibitory synaptic LTP in different brain areas, indicating that iLTP may be associated with various phenomena. The expression of iLTP is induced by the release of the neurotransmitter GABA, which is exhibited by inhibitory synapses throughout the central nervous system (CNS) and can dynamically control information flow in neural circuits [89]. Understanding various mechanisms that induce GABA release is beneficial for understanding the balance between GABA excitation and inhibition. 

**Figure 2:**
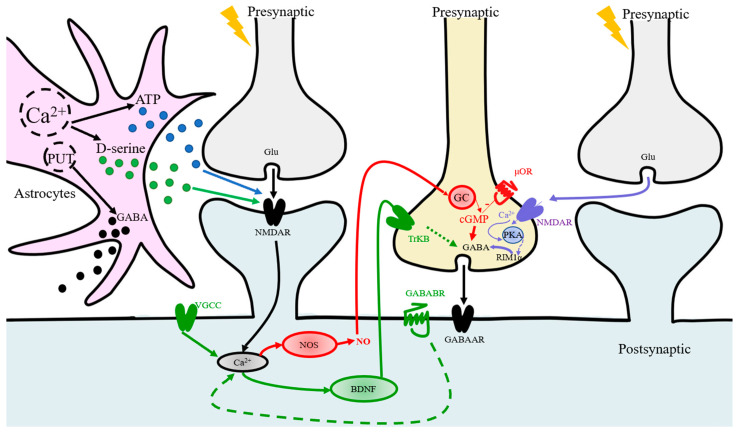
Mechanism of iLTP and the underlying mechanism of astrocyte regulation of iLTP. The red, green, and purple pathways represent the mechanism of NO-mediated long-term potentiation, BDNF-TrkB_iLTP, and NMDAR-dependent _iLTP, respectively. Astrocytes release ATP and D-serine by increasing intracellular calcium ions, which is necessary for NMDA-dependent LTP. Polyamine putrescine (PUT) is an important source of astrocyte GABA production. Significant GABA release suggests that the astrocyte Glu-GABA exchange mechanism is the key to limiting ictal discharge. This evidence may show a new mechanism for regulating iLTP.

Therefore, another important question we need to notice is the mechanism of LTP at inhibitory synapses (Figure 2) and the similarity at excitatory synapses. We have elucidated the similar mechanisms that produce LTP at inhibitory and excitatory synapses.

### 3.1. Nitric Oxide (NO)

NO is a kind of endothelium-derived relaxing factor [90], which is synthesized by NO synthase (calcium/calmodulin-dependent) with L-arginine as substrate [91]. Ca^2+^/calmodulin regulates constitutive expression types of the NOS family [92], confirming a possible connection to LTP and iLTP induction. Meanwhile, behavioral studies show that the NO/cGMP plays a role in learning and memory [93,94] because NO donors, l-Arginine, or cGMP analogs enhanced memory, whereas NOS inhibitors or genetic deletion hampered various types of memory [93].

It has been reported that, as one of the retrograde signals to maintain iLTP in GABAergic synapses in the VTA, NO first requires glutamate to activate the NMDA receptor, which increases postsynaptic calcium concentration. As a result, NO is released as a retrograde signal by NO synthase and also initiates sustained enhancement to increase cGMP levels to boost GABA release, which puts brain slices into use with NO scavengers (Mu-opioid receptors) to inhibit NO production. Single exposures to cocaine and nicotine and acute stress blocked NO-iLTP [41,95]. A combination of HFS and whole-cell recordings induced and recorded iLTP. iLTP is associated with modifying the coefficient of variation and the paired-pulse ratio of induced GABA_A_ receptors. Furthermore, IPSCs are suggested to be maintained by a sustained increase in GABA release [41].

Similarly, in a series of hippocampal neuron (CA1 and CA3) experiments [96,97,98], it was proved that NO could activate soluble guanylate cyclase (sGC), which can catalyze the conversion of GTP into cGMP after activation, increasing the level of cGMP, thereby activating cGMP-dependent protein kinase (PKG) [99]. Following that, various proteases and phosphodiesterases exert their effects to increase the release of transmitters [100,101].

As required, NO is synthesized in the cell and dendrites rather than stored in synaptic vesicles, making NO-mediated transmission different from classical forms of neurotransmission. The biological properties of NO as a gaseous molecule allow it to freely permeate biomembranes and diffuse rapidly to control synaptic transmission and plasticity.

### 3.2. BDNF-TrkB

Brain-derived neurotrophic factor (BDNF) is a protein that promotes nerve growth activity, can regulate excitatory and inhibitory transmission [88], and significantly influences the development of CNS neurons. Part of the BNDF receptors belongs to the tyrosine-related receptor kinase family (Trk), among which TrkB has the highest affinity with BDNF and is the primary functional receptor of BDNF [102]. This neurotrophin regulates synaptic function in the hippocampus by modulating presynaptic transmitter release or enhancing postsynaptic transmitter sensitivity [103]. BDNF signaling plays a role in the pathogenesis of several important diseases, including Alzheimer’s disease (AD) [104], depression, schizophrenia, and anxiety disorders [103]. Modulation of BDNF pathways could, therefore, offer a feasible strategy to treat various neurological disorders.

Gubelini et al. combined pharmacology and whole-cell recording to prove that retrograde BDNF can enhance the inhibitory function [105], whereas TrkB conductivity inhibitors do not block the inhibitory function. Induction of iLTP requires elevated postsynaptic calcium, and intracellular calcium promotes BNDF release/secretion [106]. However, different evidence indicated whether BDNF is required for LTP by combining two-photon imaging: the types of LTP at Schaffer collateral synapses selectively required BDNF. According to these findings, different presynaptic and postsynaptic modules exhibit long-term plasticity [107]. The activation of presynaptic plasticity modules, but not postsynaptic modules, depends on BDNF release from CA3 neurons. Presynaptic neurons provide BDNF, and this type of LTP requires L-type voltage-gated Ca^2+^ channel activation [107]. There is also evidence that hippocampus volume has an association with BDNF-TrkB signaling [108,109].

### 3.3. NMDAR-Dependent

NMDAR is an ion channel receptor with high calcium permeability, which can regulate neuronal activity through different neurotransmitters [110]. The key mechanism by which NMDARs participate in postsynaptic LTP induction is voltage dependence. In order to activate postsynaptic NMDARs, two conditions need to occur simultaneously. First, glutamate needs to be released and bound with the help of postsynaptic NMDARs; second, the postsynaptic membrane needs to be depolarized to remove the block of extracellular Mg^2+^. Thus, calcium influx enters the postsynaptic cell from the extracellular space through the open NMDARs, which then activates a series of signaling molecules in the postsynaptic cell, including calmodulin (CaM), protein kinase A (PKA), cyclic AMP (cAMP), immediate early genes, and enzymes that produce diffusible retrograde messengers [111]. iLTP is also present in GABAergic stellate cells (SC inhibitory synapses), and, as with LTP in excitatory synapses, it requires GABAergic terminals to activate NMDAR [112,113,114]. Stimulation with glutamatergic inputs (parallel fibers) with similar physiological activity patterns triggered a sustained increase in GABA release from stellate cells using whole-cell recordings. Moreover, in combination with extracellular recordings, enhanced inhibitory transmission reduced the firing frequency and altered the pattern of action potential activity in stellate cells. Induction of sustained increases in GABA release requires activation of NMDA receptors, and pharmacological and genetic approaches have identified presynaptic cAMP/protein kinase A (PKA) signaling and the active zone protein RIM1α as key pathways required for sustained enhancement of GABA release. Thus, a common mechanism underlies the presynaptic plasticity of excitatory and inhibitory transmissions.

Inhibitory synaptic plasticity, triggered by short- and high-frequency inhibition of the postsynaptic electrical activity of GABAergic transmission, is essentially due to an increase in postsynaptic intracellular calcium [115]. Intracellular calcium can be altered postsynaptically by various mechanisms (e.g., PKC, CaMKII, Src, and PKA [87]). These protein kinases have dual roles in LTP formation and maintenance. On the one hand, calcium ions can immediately activate them and contribute to LTP induction. On the other hand, they have an autophosphorylation function. However, the modular process for long-term potentiation induction is extremely complex and has not been completely understood yet. Future experiments using whole-cell recordings in combination with pharmacology and genetics will provide a more thorough understanding soon. 

Excitatory synapses produced homosynaptic and heterosynaptic LTP. Contrarily, iLTP mechanisms are heterosynaptic in nature, which can be induced by episodes of strong postsynaptic activity during which synapses are inactive, thereby directing any synapses that are irrelevant to heterosynaptic changes [116], and have the final goal of stimulating GABA to release into the GABA_A_R, which allows inhibitory interneurons to counteract prominent excitation and restrict neuronal activity transmission to control the output of the target neuron. It is worth mentioning that, since no synaptic stimulation is involved in the induction process due to the intracellular photolytic release of caged calcium ions and tonicity, LTP can be regarded as heterosynaptic.

In addition, as membrane clamp recordings are programmed to record synaptic functions, studying slices from inhibitory neurons or immature animals is becoming more common.

### 3.4. Glial Cells

Connecting neurons and glial cells are essential for neuroplasticity [117]. Growing evidence suggests that astrocytes are crucial for excitatory and inhibiting signaling [118]. Furthermore, gliotransmitters released by astrocytes, including ATP [117,119], D-serine [47,48], and adenosine [120], are necessary for NMDA-dependent LTP.

Importantly, glia, particularly astrocytes, bidirectionally communicate dynamically with neurons following information processing, neuronal activity, and behavior [121]. Briefly, astrocytes respond to neuronal activity and neurotransmitters by activating metabotropic receptors and releasing the gliotransmitters, which feed back to neurons [122,123]. The ATP released by astrocytes modulates synaptic transmission directly or through its metabolic product adenosine and can activate neuronal P2 receptors, P2X, and P2Y, which regulate synaptic homeostasis and plasticity [119,122]. In 2018, Adamsky et al. showed that activating astrocytic in CA1 induced LTP formation [124]. Furthermore, Stevens et al. demonstrated earliest that glial cells regulate neuronal activity by secreting D-serine [125]. Later, D-serine released from astrocytes, Ca^2+^-dependent, has been reported as closely related to LTP formation through modulating NMDA receptor function [47]. This study found that LTP formation could be blocked by clamping internal Ca^2+^ in individual CA1 astrocytes, and the blockade could be reversed by exogenous D-serine application [47]. Astrocyte–neuron communication was also related to synergism between vesicular and non-vesicular gliotransmission. Cortical astrocytes can release gliotransmitters, glutamate, and D-serine by combining SNARE-dependent exocytosis and non-vesicular mechanisms dependent on TREK-1 and Best1 channels, strongly affecting the glia-driven regulation of synaptic plasticity in hippocampus and neocortex [126]. Astrocytes have numerous large pore links. Molecular communication can travel a long distance. Neurons are divided from each other by the aquatic cleft of synapses and thus cannot interact directly with each other except through chemical communication [127]. However, astrocytes communicate extensively via large pores known as gap junctions, which may propagate molecular signaling to a long distance [128]. Moreover, this communication is enforced by polyamine spermine [127,128]. Polyamines, such as putrescine and spermine, are also gliotransmitters [118].

Putrescine and produced from putrescine GABA: some evidence pointing to an interesting mechanism. A type of gliotransmitters almost entirely stored in astrocytes: polyamines that can be released through various mechanisms. Polyamine putrescine (PUT) is an important source of astrocyte GABA production. Significant GABA release suggests that the astrocyte Glu-GABA exchange mechanism plays a key role in limiting ictal discharge [129]. In addition, polyamine spermine (SPM) is also accumulated in astrocytes but not neurons [118]. It can also modulate neuronal NMDA, AMPA, and kainate receptors [118]. This evidence may show a new mechanism for regulating iLTP.

## 4. Coordinated Plasticity of Excitatory and Inhibitory Synapses

Research on populations of glutamatergic and GABAergic synapses has previously addressed the coordinated plasticity of excitatory and inhibitory synapses. GABAergic synapses are similar to glutamatergic synapses, which can exhibit a variety of long-term plasticities at the pre- and postsynaptic levels [114,130,131]. 

**Figure 3:**
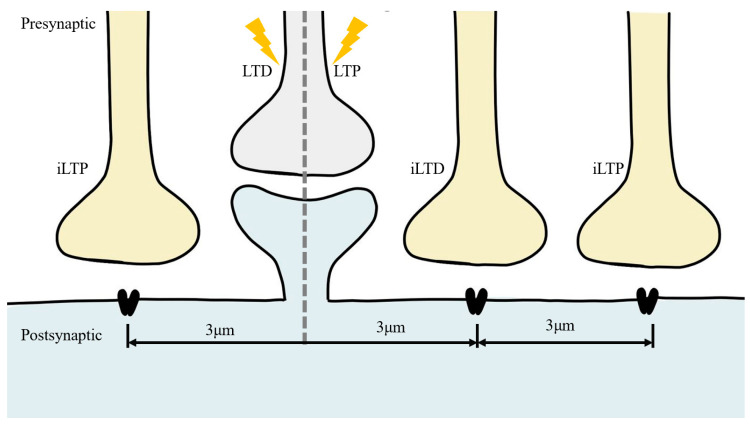
Coordinated plasticity of excitatory and inhibitory synapses [55]. LTP induction at a single glutamatergic spine leads to inhibition of nearby GABAergic inhibitory synapses (<3 μm, iLTD), while more distant synapses are enhanced (>3 μm, iLTP), and such GABA_iLTP is heterosynaptic.

Ravasenga et al. used double uncaging electrophysiology combined with single-particle tracking and pharmacology to demonstrate that induction of long-term potentiation at a single glutamatergic spine leads to inhibition of nearby GABAergic inhibitory synapses (within 3 μm, iLTD, as shown in Figure 3), while more distant synapses are enhanced (iLTP) and that such GABA_iLTP is heterosynaptic. Notably, this plastic change requires L-type calcium channels and calpain and is associated with decreased gephyrin aggregation and increased GABA_A_R mobility. Furthermore, this functional interaction is restricted to the dendritic microregions [55,132]. However, owing to the great diversity of GABAergic synaptic proteins [133] and the heterogeneity of GABAergic neurons [84], the involvement of gephyrin and the plasticity mechanisms observed here may differ depending on the specific GABAergic synaptic subtype. 

The receptor type studied above is the GABA_A_R α-subunit, which is regulated by gephyrin [134,135]. However, some evidence has also shown that not only the GABA_A_R α-subunit could regulate iLTP but also CaMKII-dependent-phospho-GABA_A_R-β3-Ser383, which promotes the accumulation of a scaffold protein (gephyrin) to induce chem-iLTP expression [136]. Additionally, metabotropic GABA_B_R [38,137,138,139,140] and other subtypes of GABAergic synapses [141] are related to the regulation of synaptic plasticity. There is evidence that iLTP induced by GABA_B_R could enhance the depression of excitatory synapses and selectively weaken excitatory input, an anticorrelated plasticity interaction [142]. Therefore, the plasticity of excitatory and inhibitory neurons appears to be interactive rather than independent. The interplay suggests that future research into the relationship between other subtypes of GABAergic synapses and receptors with excitatory neurons deserves further investigation.

## 5. Discussion

Combining electrophysiological recordings and techniques such as pharmacology, two-photon uncaging, and optogenetics can help better understand the mechanisms involved in LTP induction. Two-photon uncaging exploits the inherent optical sectioning ability of two-photon excitation to generate highly localized increases in neurotransmitter concentrations; e.g., long-term plasticity can be induced by elevated intracellular calcium concentrations generated by the photolysis of caged calcium [143,144,145]. Kano et al. used in vivo two-photon photocleavage of glutamate to find that the structure and movement of mouse cortical dendritic spines are closely related to their rapid glutamate sensing and intracellular calcium increase [146]. With the development of caged compound technology, the application of two-photon uncaging pairs in vivo to study molecular physiological processes at the single-synapse level will continue to deepen, which makes the use of two-photon glutamate uncaging to induce structural and functional LTP dendritic spines possible [147,148]. Compared to traditional single-photon imaging, two-photon imaging has a higher signal-to-noise ratio and spatial resolution, a better signal-to-noise ratio, and less tissue damage [149,150,151]. In addition to being able to monitor physiological phenomena and processes such as neural cell structure, ion concentration, cell movement, and molecular interactions at the cellular or even subcellular level, the two-photon microscope also has many precise optical manipulation functions (such as photolysis, photoactivation, phototransduction, and photodamage).

In addition, optogenetics is used to effectively express light-sensing genes in target neurons to control the activities of selected cells in highly heterogeneous tissues. Further, under the stimulation of a certain wavelength, it can selectively stimulate ions and express through special ion channels, resulting in depolarization or superization of membrane potential to excite or inhibit cells. It can control the number of ions across the membrane and change the resting potential to cause an action potential and selectively induce long-term potentiation (LTP) using optogenetics [57,152,153,154,155]. Matt Udakis et al. used optogenetics to dendrite-targeted inhibition of hippocampal CA1 pyramidal neurons and demonstrated Parvalbumin (PV) and Somatostatin (SST) inhibitory synapses have different plasticity (PV-iLTD and SST-iLTP), which are due to the employment of different signaling mechanisms (e.g., the relative timing of inhibitory and excitatory neuronal spiking) [156]. Yifeng Cheng et al. reported using optogenetics to induce LTP of the OFC→DMS pathway by exposing rats to blue light pulses through optical fibers [57]. Moreover, the latest report showed GPR173: a novel CCK receptor involved in the iLTP of CCK-INs in the cortex [58]. Ling He et al. combine optogenetics with in vivo electrophysiology to illustrate optogenetic laser stimulation of GABAergic neurons suppressed AC neuronal responses to the auditory stimulus [58]. Optogenetic technology cannot only accurately and precisely activate or inhibit specific neurons but also has high temporal and spatial resolution and reversibility benefits. An efficient combination of optogenetics and electrophysiological techniques was used to study the function of particular neuron types and circuits in LTP induction.

As shown in Table 1, most experimental designs used a single-type electrophysiological recording in combination with other forms of experimental techniques to explore a single-type synapse of LTP. The state of the circuit can change the synaptic learning principles used to induce LTP. Neuromodulators can change all network activities, the threshold and time window of plasticity induction, and even switch the plasticity marker from LTP to LTD. It is necessary to design different LTP induction protocols according to different requirements [157,158]. Therefore, the balance mechanism of excitatory and inhibitory synapses should be studied together, which may become a future design direction for electrophysiological experiments. It may also be possible to record excitatory and inhibitory neuronal activity simultaneously using field potentials and whole-cell models and simultaneously explore the activity of different types of synapses in the whole environment. The importance of excitatory synaptic LTP measured by field potentials is unquestionable; however, the role of inhibitory interneurons should not be ignored. Understanding the mechanisms of GABAergic synaptic plasticity is critical for assessing their critical role in CNS function and is fundamental to understanding the problems associated with LTP in various activities [159]. Controlling GABAergic synaptic strength is an important and growing area of research. 

It is widely accepted that LTP, resulting in synaptic modifications caused by physiological stimuli, correlates with learning and memory formation [92]. Recently, there have been increasing investigations of electrically stimulated LTP observed at inhibitory synapses in different brain areas, including the hippocampus, although most are observed at excitatory synapses previously [93]. Researchers have demonstrated that excitatory LTP provides a more effective detection, while iLTP maintains the temporal resolution of the neuronal network by using a whole-cell patch clamp [94]. It means that the excitatory LTP is dominant for short stimulation intervals due to significant increases in spike generation. Still, inhibitory LTP has an important role in preventing the degradation of this time window.

Recent studies have developed analytical methods to measure excitatory and inhibitory inputs [139] simultaneously. More specifically, both voltage clamp and current clamp recordings can be used to compute evoked LTP in vivo or in vitro [160]. For the voltage clamp measurements, it is understandable to disintegrate excitatory and inhibitory inputs directly by the linear system resolution [161]. Assuming that the regression model of the I/V curve between resting potential and the peak of activation in the voltage clamp is linear, the evoked synaptic conductance can be measured by either the linear part or the full range of the I/V curve [162]. In addition, to decrease the rectification error, researchers also utilize the polynomial regression model to the synaptic or full I/V curve. In this case, the LTP is considered the variance between the global conductance, correlated to a zero current value, and the resting conductance is measured by the tangent to the I/V curve at the resting potential. In the same way, the measurement of evoked LTP in current-clamp recordings is completed but with a current clamp mode, injecting constant currents with membrane potential near the reversal potential of inputs [160]. 

Long-term depression has been shown to reverse LTP in some synapses [163], resulting in use-dependent bidirectional changes [164]. Similarly, we can hypothesize that the increase in the population EPSP signal of LTP is excitatory enhanced due to a decrease in inhibitory signal or both. Excitation and inhibition are inextricably linked to time and space in the brain. Therefore, subtle changes in balance are linked to both neurological disease development, such as Alzheimer’s and Parkinson’s disease, and behavior [165,166,167,168]. Interrupting this dynamic balance between excitation and inhibition can significantly impact the creature’s life stability and flexibility. Therefore, these aspects deserve to be explored in greater detail in the future.

Various forms of synaptic plasticity have been shown to exist. Spike timing-dependent plasticity (STDP), a phenomenon affecting sign and magnitude of synaptic strength changes through precise spikes, is a major mechanism of the brain’s ability to learn and form new memories [169]. STDP has been demonstrated to depend on target and synaptic location and is also affected by the activity of neighboring synapses, the presence of postsynaptic calcium, presynaptic GABA inhibition, and neuromodulator dynamic adjustment [169,170,171]. Homeostatic synaptic plasticity: as a specific form of synaptic plasticity, homeostatic synaptic plasticity refers to the ability of neurons to regulate their excitability relative to network activity to maintain network homeostasis amid long-term changes in neuronal activity [172]. These different synaptic plasticities combine in complex ways to affect local circuit computation. These forms of plasticity also coexist with homeostatic mechanisms to maintain circuit function despite potentially destabilizing perturbations [89]. The coexistence of multiple forms of plasticity may reflect the hierarchical processing of information, possibly allowing the ordering of memories according to their salience [173].

Moreover, aging is thought to cause cognitive decline, which could be explained by changes in age-dependent synaptic plasticity or cellular alterations directly affecting plasticity mechanisms [174,175]. Lik-Wei Wong et al. report that the p75 neurotrophin receptor (p75NTR) may represent an important therapeutic target for limiting age-related deficits in memory and cognitive function [176]. Alexander et al. demonstrated that the perisynaptic astrocyte contraction and contraction of the processes give way to glutamate spillover. Age-dependent learning and memory impairments are possibly due to impaired synaptic plasticity [56]. Therefore, these could help better understand the age-related decline in learning and memory. Not just age but the latest evidence of Sian Lewis’ report refers to the sharing and differential expression of transcriptome-defining markers in various neurons and glia across species [177]. These results show that cells’ neurogenic potential in the hippocampal formation varies between species. 

Recent studies discovered that glial cells contribute to neuronal function by regulating extracellular K^+^ levels, leading to different CNS diseases [178]. It is reported that, in animal models of Rett syndrome (RTT), a neurodevelopmental disorder mostly due to mutations in the X-linked transcriptional regulator methyl CpG binding protein 2 (MeCP2), symptoms can be improved or prevented by re-expression of MeCP2 merely in astrocytes [179] What is more, the glia cells also have a strong impact in Huntington’s disease (HD), a neuronal disease usually associated with neuronal dysfunction and atrophy of the striatum and other brain areas. Evidence suggests that K^+^ ion channel expression was decreased in astrocytes expressing mutant huntingtin (mHTT). In contrast, the astrocyte glutamate transporter Glt1 expression was rescued by restoring the loss of K^+^ expression in glial cells, which has a strong relationship with the development of HD [180]. In pathological conditions such as Alzheimer’s disease (AD), some astrocyte neurotransmitters, such as GABA, show abnormal levels. It is observed in several studies that astrocytic GABA was elevated in the AD model [181]. In conclusion, these studies demonstrated that astrocytes are crucial for discovering the mechanisms of neurological and psychiatric diseases as the most numerous cells in the CNS. These aspects deserve to be explored in greater detail in the future.

## Data Availability

Not applicable.

## Abbreviations

LTP	Long-term potentiation
iLTP	Long-term potentiation of the inhibitory synapses
Chem-iLTP	Chemically induced iLTP
EPSP	Excitatory postsynaptic potential
IPSC	Inhibitory postsynaptic current
HP	Hippocampus
VTA	Ventral tegmental area
HFS	High-frequency stimulation
SC	Stellate cells
PKC	Protein kinase C
CaMKII	Calcium–calmodulin (CaM) dependent protein kinase II
PKA	Protein kinase A system
BDNF-TrkB	Brain-derived neurotrophic factor_ Tyrosine kinase B
NMDAR	N-methyl-D-aspartate receptor
GABA	γ-aminobutyric acid
GABA_A_R	GABA A receptor
GABA_B_R	GABA B receptor
cGMP	Cyclic guanosine monophosphate
PUT	Polyamine putrescine
SPM	Polyamine spermine
APs	Action potentials
LFPs	Local field potentials
edNEG	Electrodiffusive neuron-extracellular-glia
MEA	microelectrode array
CNS	central nervous system
Trk	tyrosine-related receptor kinase
STDP	Spike timing-dependent plasticity
MeCP2	methyl CpG binding protein 2
mHTT	mutant huntingtin
HD	Huntington’s disease
AD	Alzheimer’s disease
mHTT	mutant huntingtin
p75NTR	p75 neurotrophin receptor
ACC	Anterior cingulate cortex
oEPSCs	Optically evoked excitatory postsynaptic currents
oHFS	Optogenetic HFS
HFLS	High-frequency laser stimulation
CCKR	Cholecystokinin receptor
PUT	putrescine
LTP_CCK_	A form of activity-dependent synaptic plasticity mediated by CCK
AC	Auditory cortex
